# Skeletal Muscle Fibre-Specific Knockout of p53 Does Not Reduce Mitochondrial Content or Enzyme Activity

**DOI:** 10.3389/fphys.2017.00941

**Published:** 2017-12-04

**Authors:** Ben Stocks, Jessica R. Dent, Sophie Joanisse, Carrie E. McCurdy, Andrew Philp

**Affiliations:** ^1^School of Sport, Exercise and Rehabilitation Sciences, University of Birmingham, Birmingham, United Kingdom; ^2^Department of Human Physiology, University of Oregon, Eugene, OR, United States

**Keywords:** p53, skeletal muscle, mitochondria, metabolism, PGC-1α

## Abstract

Tumour protein 53 (p53) has been implicated in the regulation of mitochondrial biogenesis in skeletal muscle, with whole-body p53 knockout mice displaying impairments in basal mitochondrial content, respiratory capacity, and enzyme activity. This study aimed to determine the effect of skeletal muscle-specific loss of p53 on mitochondrial content and enzyme activity. Mitochondrial protein content, enzyme activity and mRNA profiles were assessed in skeletal muscle of 8-week-old male muscle fibre-specific p53 knockout mice (p53 mKO) and floxed littermate controls (WT) under basal conditions. p53 mKO and WT mice displayed similar content of electron transport chain proteins I-V and citrate synthase enzyme activity in skeletal muscle. In addition, the content of proteins regulating mitochondrial morphology (MFN2, mitofillin, OPA1, DRP1, FIS1), fatty acid metabolism (β-HAD, ACADM, ACADL, ACADVL), carbohydrate metabolism (HKII, PDH), energy sensing (AMPKα2, AMPKβ2), and gene transcription (NRF1, PGC-1α, and TFAM) were comparable in p53 mKO and WT mice (*p* > 0.05). Furthermore, p53 mKO mice exhibited normal mRNA profiles of targeted mitochondrial, metabolic and transcriptional proteins (*p* > 0.05). Thus, it appears that p53 expression in skeletal muscle fibres is not required to develop or maintain mitochondrial protein content or enzyme function in skeletal muscle under basal conditions.

## Introduction

Tumour protein p53 (p53) was initially characterised as a tumour suppressor protein (Matoba et al., 2006; Vousden and Prives, 2009; Muller and Vousden, 2013), serving to regulate cellular metabolism and proliferation (Zhou et al., 2003; Bensaad et al., 2006; Matoba et al., 2006). More recently, a functional role of p53 for *in vivo* skeletal muscle physiology has been proposed, following observations that p53 can regulate apoptosis (Saleem et al., 2009), atrophy (Fox et al., 2014), autophagy (Saleem et al., 2014), mitochondrial DNA stability (Saleem and Hood, 2013; Safdar et al., 2016), post-exercise signalling (Saleem et al., 2009, 2014), mitochondrial function (Park et al., 2009; Saleem et al., 2009, 2014; Wang et al., 2013) and endurance performance (Park et al., 2009; Saleem et al., 2009; Wang et al., 2013) within skeletal muscle.

Whole-body knockout (KO) of p53 in mice results in a deficient skeletal muscle mitochondrial phenotype (Park et al., 2009; Saleem et al., 2009), displaying reduced mitochondrial mass, mtDNA copy number, cytochrome-c oxidase enzyme activity, and state 3 respiration (Park et al., 2009; Saleem et al., 2009). As a consequence, endurance capacity and voluntary wheel running are also reduced in p53 KO mice (Park et al., 2009; Saleem et al., 2009). In comparison, oncogenic p53 mutations found in the Li-Fraumeni syndrome increase *in vivo* skeletal muscle oxidative phosphorylation in humans (Wang et al., 2013), while mitochondrial respiration and content of electron transport chain proteins is increased in primary myoblasts from Li-Fraumeni carriers and in mice carrying a p53 R712H polymorphism (Wang et al., 2013). Thus, it is clear that p53 plays an important role in mitochondrial metabolism and function.

Whilst loss of p53 impairs mitochondrial function, importantly, p53 KO mice still respond to endurance exercise training (Saleem et al., 2009). Specifically, p53 KO and WT mice display similar increases in cytochrome-c oxidase activity with training, while trained p53 KO mice exhibit no difference in electron micrograph determined subsarcolemmal mitochondrial density compared to trained WT mice (Saleem et al., 2009). This suggests that p53 is not essential for endurance exercise induced mitochondrial adaptations (Saleem et al., 2009; Safdar et al., 2016), and the functional deficits of p53 KO appear to arise in the basal (i.e., non-exercised) state.

Despite the wealth of evidence that p53 is important for whole-body metabolism and skeletal muscle mitochondrial function, determining the importance of p53 specifically in skeletal muscle cannot be ascertained from models of whole-body p53 deletion. In such models, it cannot be excluded that phenotypic differences in skeletal muscle physiology may arise as secondary defects due to dysfunction induced by the loss of p53 in other cell types. Thus, to elucidate the role of p53 specifically within skeletal muscle fibres, this study determined the effect of skeletal muscle fibre-specific loss of p53 (mKO) on mitochondrial content and enzyme activity in skeletal muscle.

## Materials and methods

### Mouse strains

The development and validation of the p53 mKO mouse has been described previously (Fox et al., 2014). Briefly, p53 mKO mice were generated by crossing homozygous p53 floxed mice (p53^f/f^; exons 2–10 of the *p53* gene are flanked by *LoxP* restriction sites) with mice expressing cre recombinase (Cre) under the control of the muscle creatine kinase (MCK) promoter. Control mice (WT) were p53^f/f^ littermates that lack the MCK-Cre transgene. All mice were on a C57BL/6 background. Eight-week old male mice were used for all experiments. Mice were housed in colony cages at 21°C with 12:12-h light-dark cycles and *ad libitum* access to standard laboratory chow (Harlan-Teklad formula 7913) and water. All animal procedures were approved by the Institutional Animal Care and Use Committee of the University of Iowa.

### Tissue collection and preparation

Muscle was obtained from young (8-weeks old), healthy mice under basal conditions. Gastrocnemius, quadriceps and triceps muscle was rapidly dissected and rinsed to remove blood and fur before being snap-frozen in liquid nitrogen. Muscle was powdered using a Cellcrusher tissue pulverizer (Cellcrusher, Co. Cork, Ireland) on dry ice and stored at −80°C prior to analysis.

### Immunoblotting

**Figure 1:**
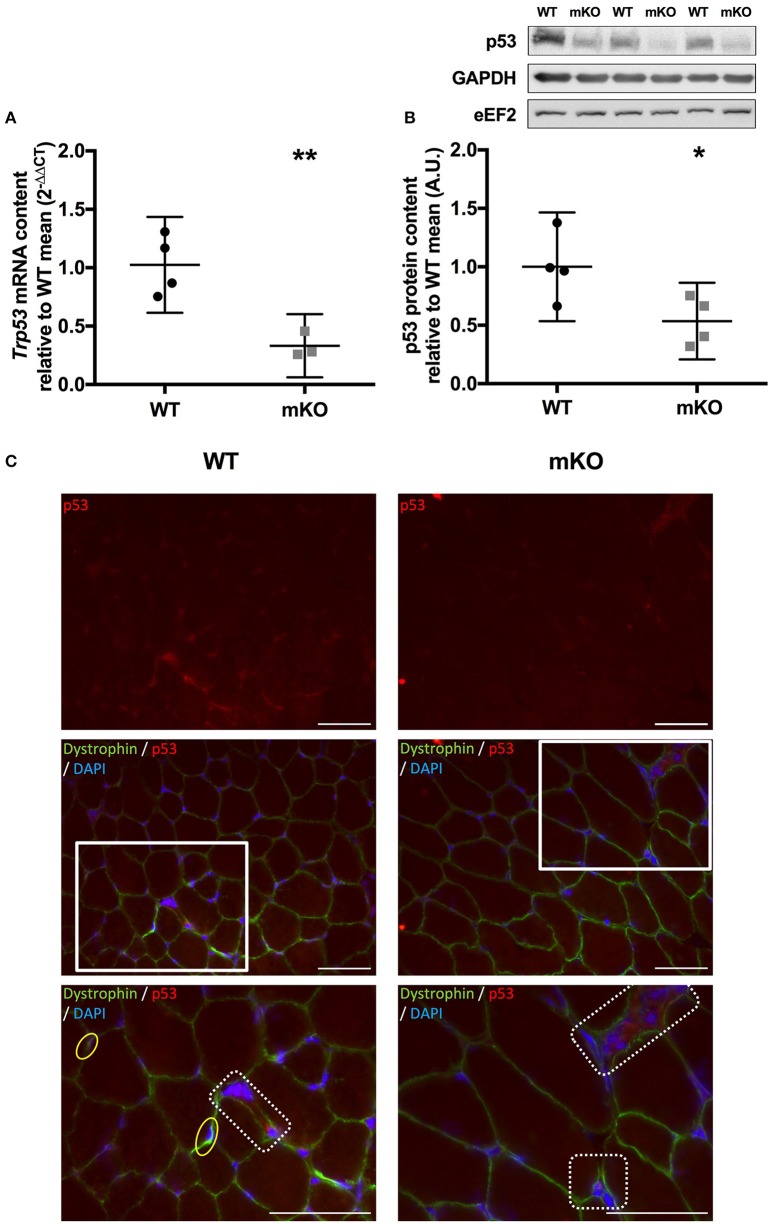
Confirmation of p53 deletion in p53 mKO mouse model. **(A)** Reduction in *Trp53* mRNA expression in quadriceps muscle of p53 mKO (grey squares) vs. WT (black circles) mice (*n* = 3–4 per group). **(B)** Reduction in p53 protein content in triceps muscle of p53 mKO mice (*n* = 4 per group). ^*^p ≤ 0.05 mKO vs. WT; ^**^p ≤ 0.01 mKO vs. WT. **(C)** Representative immunofluorescence images for WT (left column) and mKO (right column) tibialis anterior muscle. The top row represents p53 only (red), the middle row shows a composite image of p53 (red), dystrophin (green), and dapi (blue), the third row shows an enlarged image of regions highlighted with a white box in the middle row. A reduction in overall staining can be seen in mKO compared to WT. Positive regions of p53 outside of the myofibre are apparent in both WT and mKO muscle and have been highlighted in dotted boxes in the third row. Myonuclei positive for p53 are apparent only in WT muscle and have been highlighted in yellow ovals in the third row. The scaling line in each image represents 50 μM.

**Figure 2:**
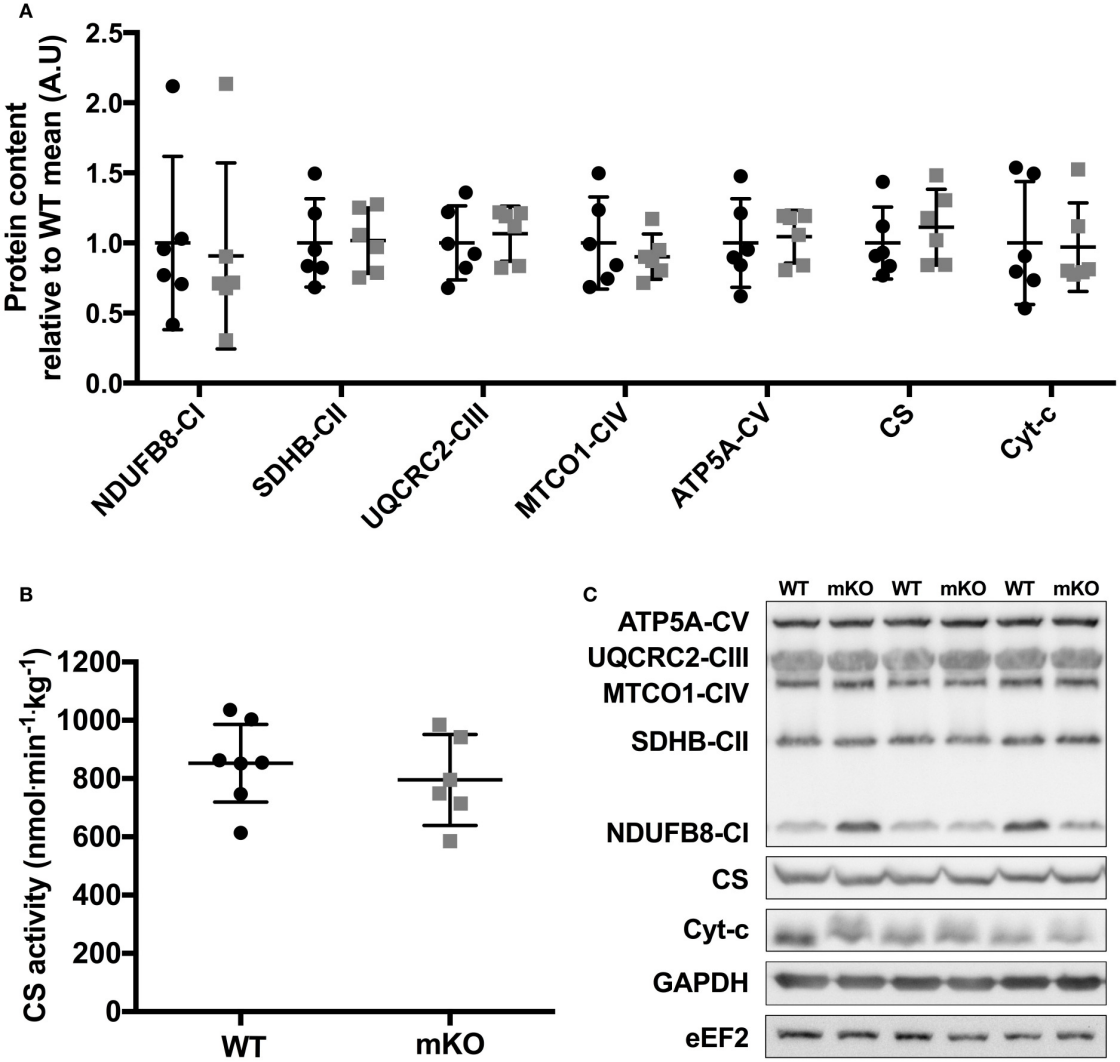
Mitochondrial OXPHOS protein content and CS enzyme activity in p53 mKO (grey squares) and WT (black circles) mice. **(A)** WT and p53 mKO mice display similar protein content of mitochondrial enzymes (complexes I-V, CS, and Cyt-c) in gastrocnemius muscle (*p* > 0.05; *n* = 6 per group). Similar data is apparent in triceps and quadriceps muscle (data not shown). **(B)** Similar CS enzyme activity in triceps muscle of p53 mKO and WT mice (*p* > 0.05; *n* = 6–7 per group). Similar data is apparent in gastrocnemius and quadriceps muscle (data not shown). **(C)** Representative immunoblot images.

**Figure 3:**
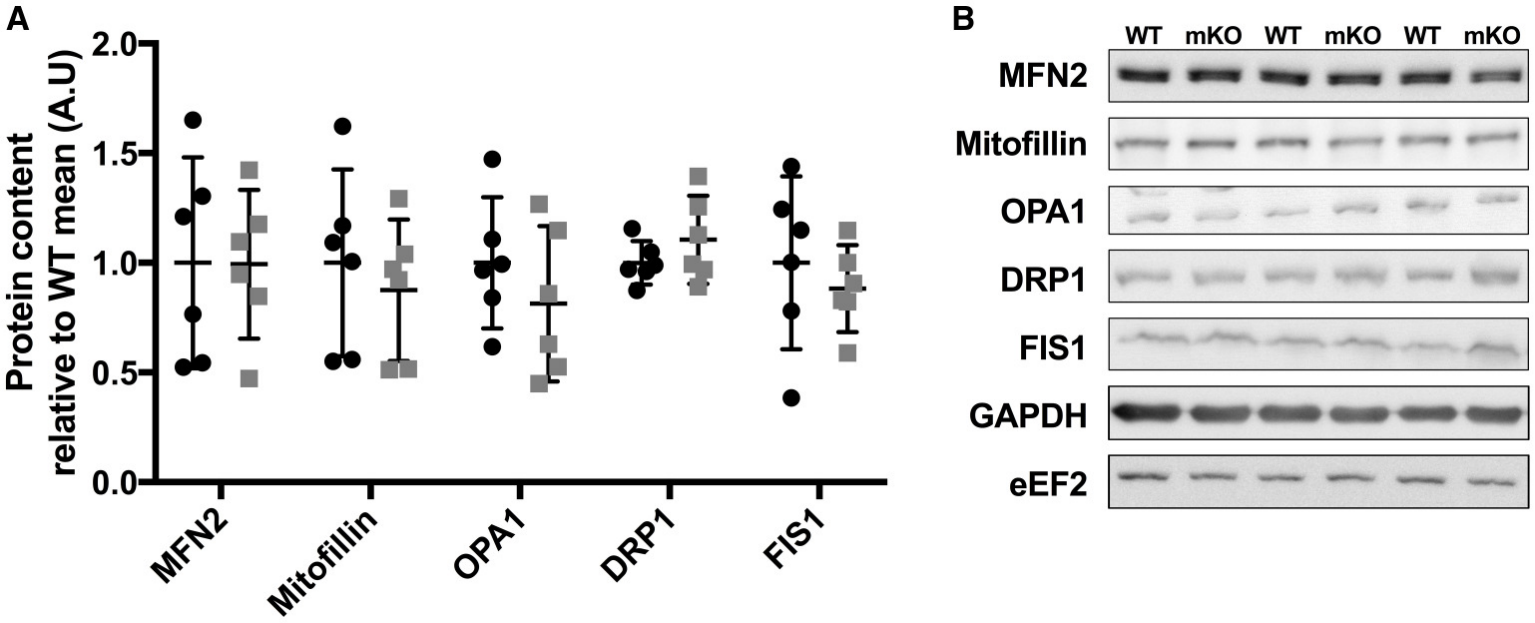
Proteins controlling mitochondrial morphology are unchanged in p53 mKO (grey squares) compared to WT (black circles) mice. **(A)** WT and p53 mKO mice display similar content of proteins regulating mitochondrial fusion (MFN2, mitofillin, OPA1) and fission (DRP1 and FIS1) in gastrocnemius muscle (*p* > 0.05; *n* = 6 per group). Similar data is apparent in triceps and quadriceps muscle (data not shown). **(B)** Representative immunoblot images.

**Figure 4:**
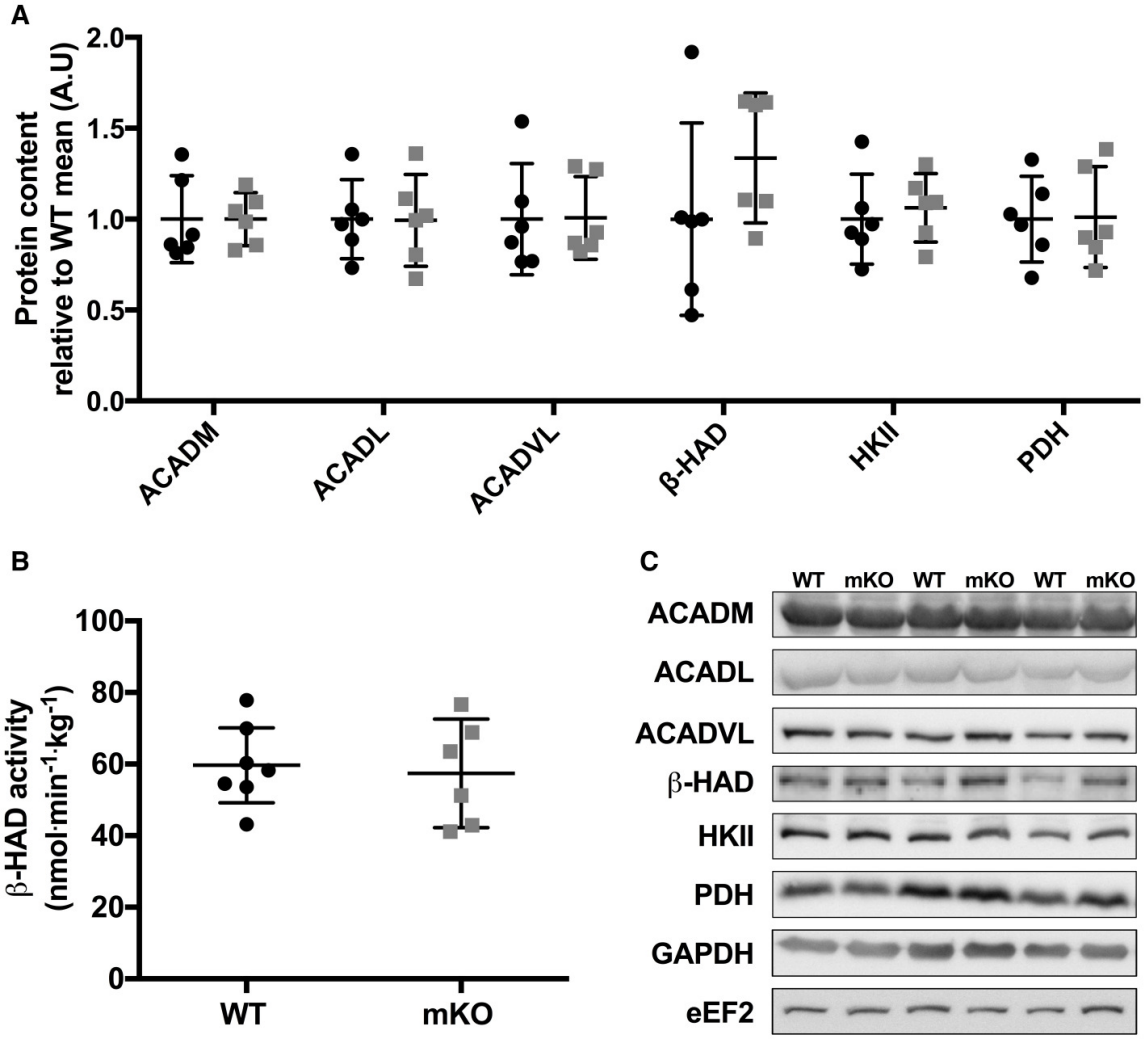
Abundance of fat and carbohydrate metabolism proteins are consistent between p53 mKO (grey squares) and WT (black circles) mice. **(A)** WT and p53 mKO mice display similar protein content of proteins involved in fatty acid (ACADM, ACADL, ACADVL, and β-HAD) and carbohydrate (HKII and PDH) metabolism in gastrocnemius muscle (*p* > 0.05; *n* = 6 per group). Similar data is apparent in triceps and quadriceps muscle (data not shown). **(B)** Similar β-HAD enzyme activity in triceps muscle of p53 mKO and WT mice (*p* > 0.05; *n* = 6–7 per group). Similar data is apparent in gastrocnemius and quadriceps muscle (data not shown). **(C)** Representative immunoblot images.

**Figure 5:**
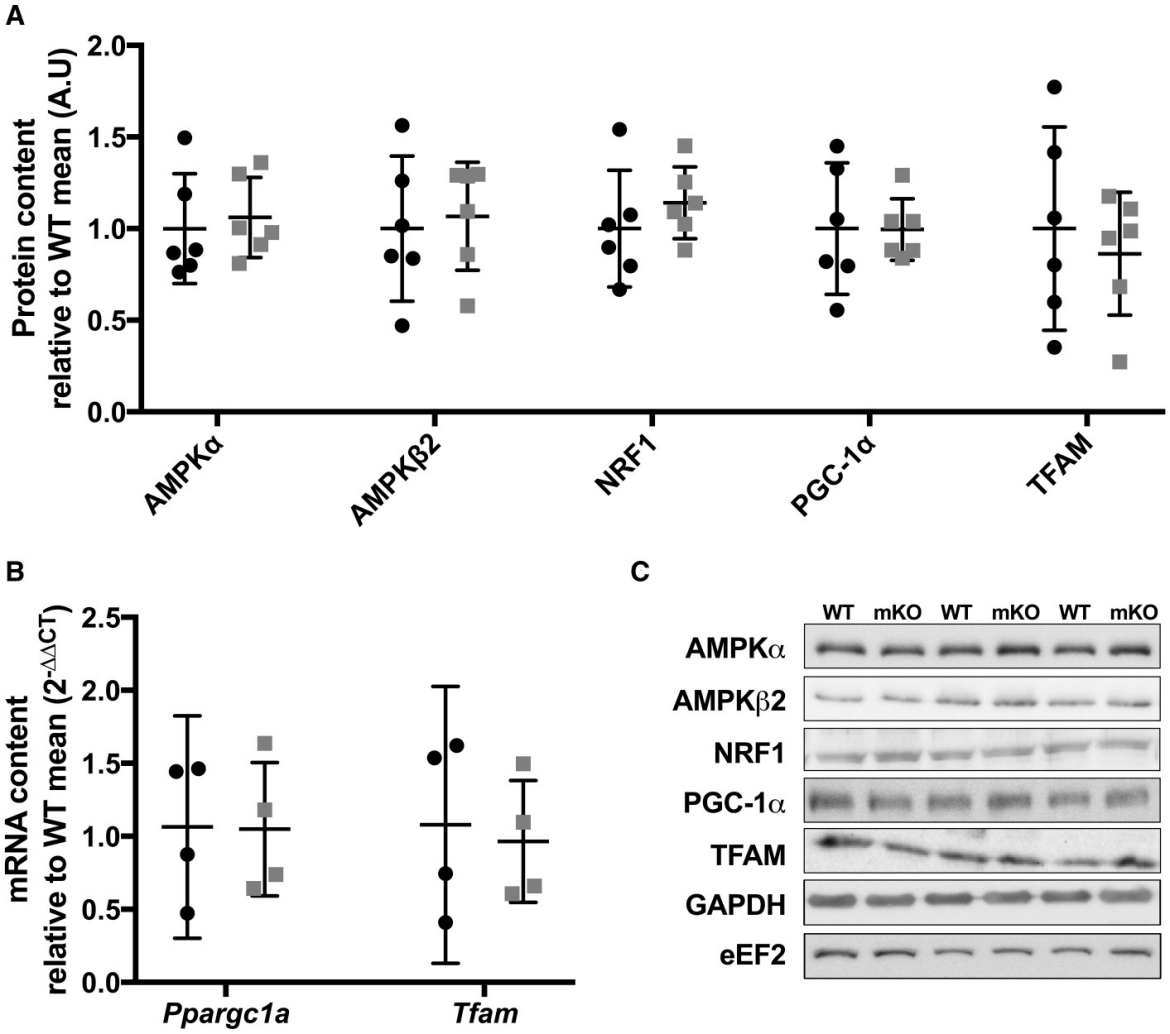
Protein content of mitochondrial biogenic signalling and transcriptional proteins in p53 mKO (grey squares) and WT (black circles) mice. **(A)** The content of proteins regulating energy sensing (AMPKα, AMPKβ2), nuclear transcription (NRF1 and PGC-1α) and mitochondrial transcription (TFAM) are comparable in gastrocnemius muscle of WT (black circles) and p53 mKO (grey squares) mice (*p* > 0.05; *n* = 6 per group). Similar results were observed in triceps and quadriceps muscle (data not shown). **(B)** The mRNA expression of *Pparg1a* and *Tfam* are similar in quadriceps muscle of WT (black circles) and p53 mKO (grey squares) mice (*p* > 0.05; *n* = 4 per group). **(C)** Representative immunoblot images.

Tissue was homogenised in a 10-fold mass excess of ice-cold sucrose lysis buffer (50 mM Tris, 1 mM EDTA, 1 mM EGTA, 50 mM NaF, 5 mM Na_4_P_2_O_7_-10H_2_O, 270 mM sucrose, 1 M Triton-X, 25 mM β-glycerophosphate, 1 μM Trichostatin A, 10 mM Nicatinamide, 1 mM 1,4-Dithiothreitol, 1% Phosphatase Inhibitor Cocktail 2; Sigma, 1% Sigma Phosphatase Inhibitor Cocktail 2; Sigma, 4.8% cOmplete Mini Protease Inhibitor Cocktail; Roche) by shaking in a FastPrep 24 5G (MP Biomedicals, Santa Ana, California, USA) at 6.0 m·s^−1^ for 80 s and centrifuging at 4°C and 8,000 g for 10 min to remove insoluble material. Protein concentrations were determined by the DC protein assay (Bio-Rad, Hercules, California, USA). Samples were boiled at 97°C for 5 min in laemmli sample buffer and an equal volume of protein (20–50 μg) was separated by SDS-PAGE on 8–12.5% gels at a constant current of 23 mA per gel. To demonstrate the loss of p53 in mKO skeletal muscle (Figure 1B), the triceps muscle from mKO and WT mice were immunoblotted. Gastrocnemius, quadriceps and triceps muscle were all immunoblotted to examine the effect of p53 mKO on the content of mitochondrial (Figures 2, 3), metabolic (Figure 4) and signalling and transcriptional (Figure 5) proteins. The presented data is from gastrocnemius muscle. Proteins were transferred on to BioTrace NT nitrocellulose membranes (Pall Life Sciences, Pensacola, Florida, USA) via wet transfer at 100 V for 1 h. Membranes were then stained with Ponceau S (Sigma-Aldrich, Gillingham, UK) and imaged to check for even loading. Membranes were blocked in 3% dry-milk in tris-buffered saline with tween (TBST) for 1 h before being incubated in primary antibody overnight at 4°C. Membranes were washed in TBST three times prior to incubation in appropriate horse radish peroxidase-conjugated secondary antibody at room temperature for 1 h. Membranes were then washed in TBST three times prior to antibody detection via enhanced chemiluminescence horseradish peroxidase substrate detection kit (Millipore, Watford, UK). Imaging and band quantification were undertaken using a G:Box Chemi-XR5 (Syngene, Cambridge, UK).

### Antibodies

All primary antibodies were used at a concentration of 1:1000 in TBST unless otherwise stated. Antibodies: 3-hydroxyacyl-CoA dehydrogenase (β-HAD; 37673), Mitofillin (110329) and MitoProfile OXPHOS antibody cocktail (110413) from abcam; cytochrome-c (Cyt-c; 556433) from BDPharmingen; 5′ AMP-activated protein kinase alpha (AMPKα; 2603), AMPKβ2 (4148), dynamin-1-like protein (DRP1; 8570), glyceraldehyde 3-phosphate dehydrogenase (GAPDH; 2118; 1:5000), eukaryotic elongation factor 2 (eEF2; 2332), hexokinase II (HKII; 2867), mitofusin 2 (MFN2; 9482), pyruvate dehydrogenase (PDH; 2784) and p53 (2524; 1:1000 in 5% non-fat dry milk in TBST) from Cell Signaling Technologies; Dynamin-like 120 kDa protein (OPA1; CPA3687) from Cohesion Biosciences; peroxisome proliferator-activated receptor gamma coactivator 1-alpha (PGC-1α; ST1202) from EMD Millipore; citrate synthase (CS; SAB2701077), mitochondrial fission protein 1 (FIS1; HPA017430; 1:500), and mitochondrial transcription factor A (TFAM; SAB1401383) from Sigma-Aldrich; Acyl-CoA dehydrogenase medium chain (ACADM; 1:2000), Acyl-CoA dehydrogenase long chain (ACADL; 1:5000), Acyl-CoA dehydrogenase very long chain (ACADVL; 1:5000) were kind gifts from Prof Jerry Vockley, University of Pittsburgh, USA. Secondary antibodies were used at a concentration of 1:10000 in TBST. Anti-rabbit (7074) and anti-mouse (7076) antibodies were from Cell Signaling Technology; anti-chicken (PA1-28798) was from Thermo Scientific.

### Immunofluorescence

Tibialis anterior skeletal muscle was embedded and frozen in tissue freezing medium (Triangle Biomedical, Durham, North Carolina, USA) in nitrogen-cooled isopentane, muscle samples were stored at −80°C. Skeletal muscle cross sections (7 μM) of WT and mKO tibialis anterior muscle were prepared using a microtome blade (Bright 5040, Bright Instrument Company limited, Huntingdon, England). Muscle sections of WT and mKO were collected onto the same uncoated glass slides and stored at −80°C until future analysis. Samples were thawed and fixed in an acetone:methanol (1:1) solution at −20°C then washed in PBS. Samples were permeabilised in a 0.2% TritonX-100 solution for 10 min, then washed in PBS and followed by a 30 min incubation in 5% normal goat serum (Invitrogen, UK) prepared in 1% BSA. Samples were then incubated in p53 primary antibody (SCBT sc-6243, rabbit, polyclonal; 1:20) prepared in 1% BSA overnight at 4°C. Slides were washed in PBS and incubated for 2 h in goat anti rabbit IgG Alexa 594 (ThermoFisher Scientific Inc. Waltham, MA, USA; 1:200) secondary antibody. Slides were then stained for dystrophin (DSHB, MANDYS1, mouse, monoclonal; 1:200) for 2 h, washed in PBS and treated with goat anti mouse IgG2a Alexa 488 (ThermoFisher Scientific Inc. Waltham, MA, USA 1:200) secondary antibody for 2 h. Nuclei were labelled with DAPI (4′,6-diamidino-2-phenylindole) (1:1000, Sigma-Aldrich, UK), prior to cover slipping with 20 μL Mowiol® 4-88 (Sigma-Aldrich, UK). Appropriate secondary antibody only control slides were used to ensure the specificity of the p53 antibody and that no apparent staining of dystrophin was visualized using the 540–580 nm excitation filter in which p53 was detected. Slides were visualised using a Nikon E600 widefield microscope with a 40 × 0.75 numerical aperture objective. Images were captured under three colour filters using a SPOT RT KE colour three shot CCD camera (Diagnostic Instruments Inc., MI, USA), illuminated by a 170 W Xenon light source. All images were captured using the same exposure time and gain for p53 staining in both WT and mKO muscle sections.

### Citrate synthase and 3-hydroxyacyl-coA dehydrogenase enzyme activity assays

Tissue was homogenised in a 10-fold mass excess of ice-cold sucrose muscle homogenisation buffer (Spinazzi et al., 2012) by shaking in a FastPrep 24 5G (MP Biomedicals) at 6.0 m^.^s^−1^ for 80 s. Protein concentrations were determined by the DC protein assay (Bio-Rad, Hercules, California, USA). Gastrocnemius, quadriceps, and triceps muscle were all analysed for enzyme activity with data presented from triceps muscle. An equal volume of protein (10 μg for CS, 20 μg for β-HAD) was loaded onto 96-well microtiter plates in triplicate. For CS, 10 μL of sample was diluted in 235 μL of reaction buffer [64 mM TRIS pH 8.0, 0.13 mM 5,5-dithio-bis-(2-nitrobenzoic acid), 0.13 mM acetyl CoA]. Five microlitres of 5 mM oxaloacetate was added to start the reaction and absorbance was read at 412 nm for 3 min in a FLUOstar Omega microplate reader (BMG Labtech, Aylesbury, UK). For β-HAD, 15 μL of sample was diluted in 230 μL of reaction buffer (68 mM TRIS pH 8.0, 270 mM NADH, 270 mM EDTA, 270 mM Triton X-100). Five mictolitres of 5 mM aceto-acetyl CoA was added to start the reaction and absorbance was read at 340 nm for 12 min. Enzyme activity in nmol^.^min^−1.^mg^−1^ was determined from absorbance using the equation presented in Spinazzi et al. (2012) corrected for differences in pathlength.

### RNA isolation and reverse transcription—polymerase chain reaction

RNA was extracted from quadriceps muscle by Tri reagent (Sigma Aldrich, Gillingham, UK) and purified on Reliaprep spin columns (Promega, Madison, Wisconsin, USA) using the manufacturer's instructions. RNA concentrations were determined using the LVis function of the FLUOstar Omega microplate reader. RNA was diluted to 400 μg/μL and reverse transcribed to cDNA using the RT^2^ First Strand kit (Qiagen, Manchester, UK). RT-PCR analysis of mRNA content was performed in singleton by using custom designed 384-well RT^2^ PCR Profiler Array (Qiagen) and RT^2^ SYBR Green Mastermix (Qiagen) on a CFX384 Real-Time PCR Detection System (Bio-Rad). 2.8 ng of cDNA was added to each well. The absence of genomic DNA, the efficiency of reverse-transcription and the efficiency of the PCR assay were assessed on each plate and conformed to the manufacturers limits in each case. Relative mRNA expression was determined using the 2^−ΔΔCT^ method (Livak and Schmittgen, 2001) with the mean C^T^ value for *Gapdh, Actb, Hsp90ab1*, and *B2m* used as an internal control.

### Statistics

Difference between genotypes was determined by independent *t*-tests using the Statistical Package for the Social Sciences (SPSS) version 22.0. Data is presented as means with 95% confidence intervals. Statistical significance was accepted as *p* ≤ 0.05.

## Results

### Confirmation of p53 deletion in the p53 mKO mouse model

In agreement with the previous characterisation of the p53 mKO model (Fox et al., 2014) we observed an ~70% reduction in *Trp53* mRNA (Figure 1A), while an ~60% reduction in p53 protein content (Figure 1B) in skeletal muscle tissue from p53 mKO mice was also apparent. Immunofluorescence staining of WT and mKO tibialis anterior muscle cross sections indicates an overall reduction in p53 staining and a loss of p53-positive myonuclei (Figure 1C). However, non-myofibrillar p53 staining is apparent in both WT and mKO muscle suggesting that the remaining p53 apparent in immunoblots of mKO muscle likely reflects expression from non-muscle fibre cells resident within skeletal muscle tissue.

### Mitochondrial content and enzyme activity is maintained in p53 mKO

Despite deletion of p53 in skeletal muscle fibres, the content of proteins within the electron transport chain were similar between p53 mKO and WT littermates (Figure 2A). Maximal activity of citrate synthase (Figure 2B), a strong correlate and validated surrogate of mitochondrial content (Larsen et al., 2012), as well as cytochrome-c and citrate synthase protein content (Figure 2A) were also maintained in p53 mKO mice. In addition, proteins involved in the regulation of mitochondrial fission (DRP1, Fis1) and fusion (MFN2, mitofillin, OPA1) were also unaffected by p53 mKO (Figure 3). Thus, mitochondrial protein content and enzyme activity in skeletal muscle is maintained following muscle fibre-specific deletion of p53.

### Loss of p53 does not alter regulators of substrate metabolism in skeletal muscle

p53 mKO did not reduce the content of proteins involved in fatty acid transport and metabolism (ACADM, ACADl, ACADVL, β-HAD) or carbohydrate metabolism (HKII, PDH; Figure 4A). Furthermore, the activity of the mitochondrial-localised fatty acid metabolic protein β-HAD was unaffected by p53 mKO (Figure 4B).

### Proteins controlling energy sensing and mitochondrial gene expression are unaffected by p53 mKO

Whole-body p53 KO mice exhibit reduced skeletal muscle mRNA and protein expression of TFAM (Park et al., 2009) and protein content of PGC-1α (Saleem et al., 2009), while p53 has been implicated in the transcriptional control of AMPK subunits (Feng et al., 2007). Therefore, we studied the protein content of PGC-1α, TFAM, AMPKα, AMPKβ2, or NRF1 within p53 mKO skeletal muscle. In contrast to whole-body p53 KO mice, p53 mKO does not reduce the protein content of PGC-1α, TFAM, AMPKα, AMPKβ2, or NRF1 within skeletal muscle (Figure 5A) or the mRNA expression of PGC-1α or TFAM (Figure 5B).

### Gene expression of proteins involved in skeletal muscle function and metabolism

**Figure 6:**
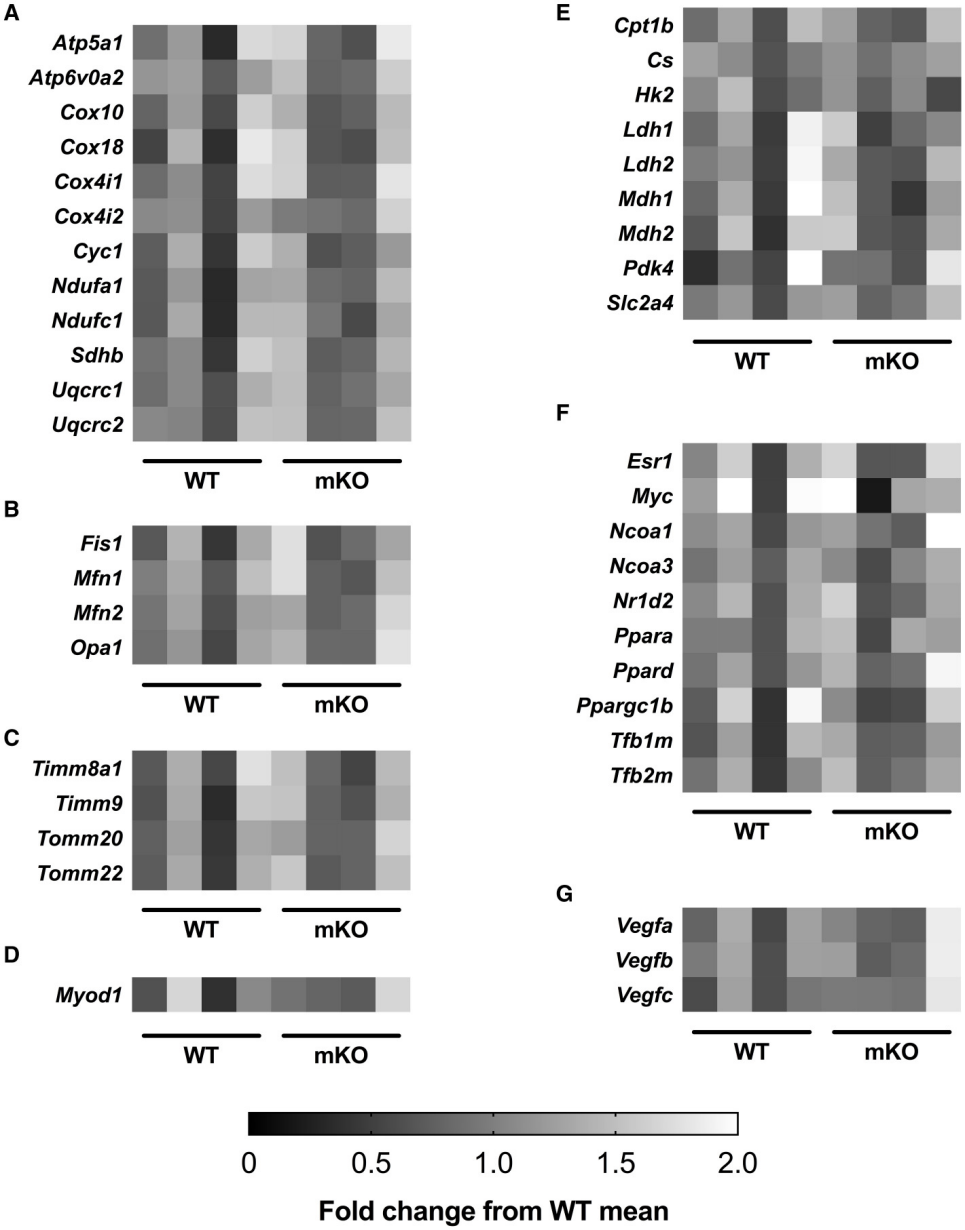
p53 mKO and WT mice display similar mRNA expression of **(A)** electron transport chain proteins and proteins involved in regulating **(B)** mitochondrial morphology, **(C)** mitochondrial protein transport, **(D)** muscle development, **(E)** carbohydrate and fatty acid metabolism, **(F)** transcription and **(G)** angiogenesis. Each column represents the mRNA expression from the quadriceps muscle of a single mouse (*p* > 0.05; *n* = 4 per group).

p53 mKO did not affect the mRNA expression of a sub-set of electron transport chain, mitochondrial morphology, mitochondrial transport, carbohydrate and fatty acid metabolism, transcription, angiogenic, or muscle development genes (Figure 6).

## Discussion

We report that muscle-specific deletion of p53 does not reduce mitochondrial protein content or enzyme activity within skeletal muscle. This is in contrast to previous research from whole-body p53 KO mice which reported a reduction in mitochondrial mass, mtDNA copy number, cytochrome-c oxidase enzyme activity, and state 3 respiration (Park et al., 2009; Saleem et al., 2009). Given these discrepancies, we therefore interpret the data to suggest that the decrement in skeletal muscle mitochondrial function in whole-body p53 KO mice is a secondary consequence of an adverse phenotype of this mouse model, rather than due the loss of p53 *per se* in skeletal muscle fibres.

p53 has been implicated in metabolic control within numerous cell types (Zhou et al., 2003; Bensaad et al., 2006; Matoba et al., 2006). Indeed, p53 can exert direct or indirect transcriptional control over various metabolic and mitochondrial biogenic proteins in some cell types [e.g., PGC-1α (Saleem et al., 2009), TFAM (Park et al., 2009; Saleem et al., 2009), CO1 (Okamura et al., 1999), SCO2 (Matoba et al., 2006), AMPKβ (Feng et al., 2007), TIGAR (Bensaad et al., 2006), and GLUT4 (Schwartzenberg-Bar-Yoseph et al., 2004)]. However, our data suggests that in skeletal muscle, p53 is not required to maintain mitochondrial content and function. For example, the maximal activities of citrate synthase and the protein content of electron transport chain proteins were maintained in p53 mKO mice. In addition, the content of proteins controlling mitochondrial morphology, substrate utilisation, energy sensing, and transcription were comparable between p53 mKO and WT mice. The muscles examined within this study were, however, all predominantly made up of fast-fibre types and it cannot be excluded that a different phenotype may be apparent in predominantly slow-fibre type muscles. Nonetheless, this data does suggest a normal metabolic phenotype of p53 mKO mice under basal conditions and is consistent with a similar skeletal muscle fibre-type distribution and diameter previously reported in p53 mKO mice (Fox et al., 2014).

Unimpaired mitochondrial biogenesis in skeletal muscle of p53 mKO mice is in contrast to data published from whole-body p53 KO mice (Saleem et al., 2009). Why whole-body p53 KO mice exhibit mitochondrial defects within skeletal muscle while p53 mKO mice do not is unclear. One explanation for the divergent phenotypes may be differences in the timing of p53 deletion in skeletal muscle between the two models. In the mKO mice examined here Cre was expressed under the MCK promoter and as such p53 would not be deleted until ~13 days into embryonic development (Lyons et al., 1991), whereas p53 is absent throughout the entirety of embryonic development in the germline deletion of the whole-body KO mice. Importantly, p53 is highly expressed in mouse embryos from embryonic day 8.5–10.5 (Schmid et al., 1991). During myogenesis, p53 plays an important role in inducing differentiation (Porrello et al., 2000; Cam et al., 2006); a period of intense mitochondrial biogenesis (Remels et al., 2010). p53^−/−^ myoblasts and C2C12 myoblasts treated with dominant-negative p53 inhibitors display impaired myosin heavy chain induction during differentiation, potentially due to reduced expression of the muscle differentiation controlling proteins retinoblastoma protein (RB) and MCK (Porrello et al., 2000; Cam et al., 2006). Although direct evidence for a lack of mitochondrial development during differentiation in p53^−/−^ myoblasts is lacking, mitochondrial deficits may arise in the whole-body KO mice due to a lack of p53 during this developmental period, while conversely p53 is expressed in skeletal muscle of the embryonic p53 mKO mice during this stage. Additionally, a loss of p53 in satellite cells in whole-body KO mice may impair satellite cell differentiation into myocytes (Porrello et al., 2000) during adulthood and therefore the continued regeneration and maintenance of healthy skeletal muscle (Relaix and Zammit, 2012). Thus, the loss of p53 in one or multiple cell type(s) other than muscle fibres may contribute to a decrement in mitochondrial function within skeletal muscle.

Several differences are also apparent in the expression of mitochondrial biogenic transcription factors/co-factors between the whole-body KO and mKO models. Park et al. (2009) demonstrated that p53 interacts with the TFAM gene in C2C12 myoblasts and that TFAM expression is reduced in the soleus muscle of whole-body p53 KO mice. Furthermore, PGC-1α protein content was decreased in p53 KO mice examined by Saleem et al. (2009), although this contradicts similar PGC-1α protein content found between WT and p53 KO mice by Park et al. (2009). In the p53 mKO mice studied here there was no deficit in TFAM or PGC-1α protein or mRNA expression, which may explain the normal mitochondrial phenotype exhibited by p53 mKO mice. Alternatively, given the developmental nature of the mKO model, other regulators of mitochondrial biogenesis (Perez-Schindler and Philp, 2015) may be compensating for the loss of p53 in skeletal muscle, however exploration of all these pathways is outside the scope of this investigation. Thus, an inducible muscle-specific knockout model is still required to determine whether p53 is important for maintaining mitochondrial function in mature skeletal muscle.

Finally, the whole-body p53 KO mouse may not be a healthy model. Whole-body p53 KO mice exhibit reduced voluntary wheel running (Saleem et al., 2009), which if recapitulated as lower spontaneous physical activity in non-exercised mice may explain the reduced mitochondrial function. Furthermore, whole-body p53 KO mice develop spontaneous cancers at an early age (Donehower et al., 1992), something that is often concomitant with cachexia and impaired muscle function. Nonetheless, utilising a muscle fibre-specific knockout model allows us, and others (Safdar et al., 2016), to assess p53 function within skeletal muscle without the complications of potential secondary defects associated with loss of p53 in other cell types.

While p53 within skeletal muscle fibres does not appear critical for developing or maintaining mitochondrial content within skeletal muscle of young healthy mice in the basal state, p53 does clearly play a role within skeletal muscle in other physiological contexts. For example, induction of p53 contributes to immobilisation-induced atrophy by orchestrating transcription of *Cdkn1a*/p21 and possibly other genes that promote muscle fibre atrophy, whereas p53 mKO mice are protected from atrophy (Fox et al., 2014). p53 is also important in maintaining mitochondrial DNA stability and mediates exercise-induced mitochondrial biogenesis in PolG KO mice, a model of accelerated mitochondrial mutations (Safdar et al., 2016). Thus, it is likely that the importance of p53 may become apparent during ageing. Furthermore, it remains to be determined whether p53 mKO mice respond to endurance exercise training although whole-body p53 KO mice are uninhibited in this respect (Saleem et al., 2009), which would indicate that p53 mKO would also be responsive to endurance exercise training.

Our data suggests that muscle fibre-specific deletion of p53 does not impair mitochondrial protein content or enzyme activity in skeletal muscle. This is evidenced by similar content of proteins within the electron transport chain and proteins regulating mitochondrial morphology, substrate utilisation, energy sensing, and transcription between WT and p53 mKO mice. In addition, there were no deficits in the activity of the mitochondrial enzymes CS and β-HAD in p53 mKO mice. While p53 likely has other roles in skeletal muscle physiology, from this data it does not appear that skeletal muscle p53 is important for developing or maintaining mitochondrial content in young healthy mice.

## Author contributions

AP and BS conceived the study, analyzed and interpreted data, and wrote the manuscript. BS, JD, SJ, and CM contributed to acquisition of data. All authors revised the work for critical intellectual content and approved the final manuscript.

### Conflict of interest statement

The authors declare that the research was conducted in the absence of any commercial or financial relationships that could be construed as a potential conflict of interest.

## Abbreviations

ACADM: Acyl-CoA dehydrogenase medium chain
ACADL: Acyl-CoA dehydrogenase long chain
ACADVL: Acyl-CoA dehydrogenase very long chain
AMPK: 5′ AMP-activated protein kinase alpha
ATP5A: ATP synthase subunit alpha
β-HAD: 3-hydroxyacyl-CoA dehydrogenase
Cre: Cre recombinase
CS: Citrate synthase
Cyt-c: Cytochrome-c
DRP1: Dynamin-1-like protein
eEF2: Eukaryotic elongation factor 2
FIS1: Mitochondrial fission protein 1
GAPDH: Glyceraldehyde 3-phosphate dehydrogenase
HKII: Hexokinase II
KO: Knockout
MCK: Muscle creatine kinase
MFN2: Mitofusin 2
mKO: Muscle fibre-specific knockout
MTCO1: Cytochrome c oxidase subunit I
NDUFB8: NADH:Ubiquinone Oxidoreductase Subunit B8
NRF1: Nuclear respiratory factor 1
OPA1: Dynamin-like 120 kDa protein
PDH: Pyruvate dehydrogenase
PGC-1α: Peroxisome proliferator-activated receptor gamma coactivator 1-alpha
P53: Tumour protein p53
SDHB: Succinate Dehydrogenase Complex Iron Sulphur Subunit B
TFAM: Mitochondrial transcription factor A
UQCRC2: Ubiquinol-Cytochrome C Reductase Core Protein II.
